# Minimum Salaries Recommended for Fever Nurses

**Published:** 1921-01-22

**Authors:** 


### Minimum Salaries Recommended for Fever Nurses.

The following scale of *minimum* salaries for fever nurses is recommended by the Council of the Fever Nurses' Association:—

(1) Probationers and Junior Assistant Nurses.—First year, £45; second year, £50.

(2) Staff Nurses.—£60 per annum, rising by annual increments of £5 to £80. (N.B.—If a general-trained nurse obtains a fever certificate she shall receive an increase of £10 per annum.)

(3) Sisters.—(*a*) General trained, and holding a recognised fever certificate, £90, rising by annual increments of £10 to £120. (*b*) General trained, but not holding a fever certificate, £80, rising by increments of £5 per annum to £100. (N.B.—On obtaining their fever certificates sisters should automatically pass into Class (*a*).

(4) Night Superintendents and Home Sisters.—£100 per annum, rising by £10 annually to £150. (N.B.—A sister on the maximum salary in Class (*a*), who takes a position as night superintendent or home sister, is to be paid an additional salary at the rate of £20 per annum.)

(5) Assistant Matrons.—£120 to £170 per annum, by £10 annually.

(6) Matrons—Of a hospital containing up to 25 beds, £100 per annum; 25 to 50 beds, £120 per annum; 50 to 100 beds, £150 per annum; 100 to 200 beds, £200 to £250 per annum; 200 to 300 beds, £225 to £275 per annum; 300 to 400 beds, £250 to £300 per annum; 400 to 500 beds, £300 to £350 per annum; above 500 beds, £350 to £450 per annum (by £10 annually).

When the maximum salary has been obtained in Classes (3), (4), and (5) it is recommended that a long-service increase of pay should be given of £10 after five years' service, £20 after ten years' service.

We give on p. 393 the reasons which have led the Council of the Fever Nurses' Association to recommend the above salaries.

